# Effects of biological sex and pregnancy in experimental autoimmune encephalomyelitis: It’s complicated

**DOI:** 10.3389/fimmu.2022.1059833

**Published:** 2022-11-28

**Authors:** Pamela A. McCombe, Judith M. Greer

**Affiliations:** UQ Centre for Clinical Research, The University of Queensland, Brisbane, QLD, Australia

**Keywords:** experimental autoimmune encephalomyelitis, biological sex, pregnancy, sex chromosomes, sex hormones, central nervous system

## Abstract

Experimental autoimmune encephalomyelitis (EAE) can be induced in many animal strains by inoculation with central nervous system antigens and adjuvant or by the passive transfer of lymphocytes reactive with these antigens and is widely used as an animal model for multiple sclerosis (MS). There are reports that female sex and pregnancy affect EAE. Here we review the effects of biological sex and the effects of pregnancy on the clinical features (including disease susceptibility) and pathophysiology of EAE. We also review reports of the possible mechanisms underlying these differences. These include sex-related differences in the immune system and in the central nervous system, the effects of hormones and the sex chromosomes and molecules unique to pregnancy. We also review sex differences in the response to factors that can modify the course of EAE. Our conclusion is that the effects of biological sex in EAE vary amongst animal models and should not be widely extrapolated. In EAE, it is therefore essential that studies looking at the effects of biological sex or pregnancy give full information about the model that is used (i.e. animal strain, sex, the inducing antigen, timing of EAE induction in relation to pregnancy, etc.). In addition, it would be preferable if more than one EAE model were used, to show if any observed effects are generalizable. This is clearly a field that requires further work. However, understanding of the mechanisms of sex differences could lead to greater understanding of EAE, and suggest possible therapies for MS.

## Introduction

The biological sex and the features of sexual dimorphism in mammals are determined by the complement of X and Y chromosomes (1), which evolved many millions of years ago with the development of sexual reproduction (2). Sexual dimorphism occurs not only in differences in external appearances, but also in internal organs and biological functions. There are thought to be sex differences in all cells of the body (3). In mice, it has been reported that sex has an impact on ~57% of quantitative traits (i.e. continuous measurements such as body weight) and on 10% of qualitative traits (i.e. categorical measurements such as normal head shape) (4). Aside from sex differences due to effects of genes on the sex chromosomes, many of the physiological effects of sex differences are due to sex hormones (5, 6).

Many autoimmune diseases show sexual dimorphism in incidence, prevalence, and features of disease (7). One such autoimmune disease is multiple sclerosis (MS), an inflammatory and demyelinating disorder of the central nervous system (CNS). More than 80% of people initially develop a relapsing-remitting form of MS (RR-MS), whereas the remainder develop a form of disease that is progressive from the outset (primary progressive (PP)-MS). RR-MS is approximately three times more prevalent in females than males, whereas the ratio of females to males with PP-MS is closer to 1:1 (8, 9). There is also a marked effect of pregnancy on the course of MS (10, 11). There are a multitude of potential mechanisms underlying these sex differences in humans, many of which currently remain unexplored.

It has also been reported that biological sex and pregnancy can modulate the outcomes of experimental autoimmune encephalomyelitis (EAE), an animal model widely used in research on MS. In this review, we focus on how biological sex and pregnancy influence the incidence, severity, and clinical course of EAE. We conclude that there is much variability in these effects between different EAE models, suggesting that many intrinsic and extrinsic modifiers make this a much more nuanced topic than sometimes portrayed. Nevertheless, understanding how these effects occur in the animal model could point to strategies that might modify the course of MS.

## Overview of EAE

EAE developed from investigations into the cause of paralysis following anti-rabies vaccination, which used vaccines produced from desiccated spinal cords of rabies-infected rabbits. The first description of EAE in the English language literature appeared in 1933 by Rivers, Sprunt and Berry (12). The original forms of EAE were induced by multiple inoculations with emulsions of CNS tissue. Modern forms of EAE came about with the introduction of adjuvants (13). EAE can be actively induced in a range of animal species by injection of CNS antigens emulsified in adjuvant or by adoptive transfer of T cells reactive to such antigens (14–17). EAE is now most commonly induced with peptides of myelin oligodendrocyte glycoprotein (MOG), myelin basic protein (MBP) or myelin proteolipid protein (PLP), although a wide range of CNS antigens, including homogenates of brain and spinal cord, can be used (18).

Soon after its first description, some of the pathological changes in the CNS in EAE were noted to be similar to those of MS (13). However, EAE differs from MS in needing to be induced, rather than occurring spontaneously (with the exception of a small number of transgenic mouse models in which the majority of the T cells and/or B cells in the mice have receptors specific for myelin antigens (19, 20)). There are also marked differences in clinical and some of the pathological features of disease, compared to MS (21), although, similar to MS, the pathological features of EAE always include some degree of neuroinflammation and usually some demyelination and some degree of axonal injury and loss (22–25).

Nowadays, EAE is most frequently induced in mice and rats, although it can also be induced in non-human primates (26–31), guineas pigs (32–34), rabbits (35, 36), hamsters (37), dogs (38) sheep (39, 40) or opossums (41). Within species, animal strains differ in their susceptibility to EAE and the clinical and pathological features of disease (28, 31, 34, 36, 39). It should also be noted that many factors in addition to the animal strain and encephalitogen can affect the development of EAE, including the source of the animals and the sterility of the environment in which they are housed (which affects the gut microbiome), the age of the animals, the adjuvant(s) in which the encephalitogen is emulsified and the way in which emulsions are prepared and injected, and whether or not pertussis toxin is also given to the animals (commonly used in mice, but rarely in other species). Several detailed standard protocols for induction of actively- or passively-induced EAE have been published (42–45), but there are many variations on a theme in the literature.

## Effects of the sex of experimental animals on incidence and clinical features of EAE

Interpreting whether or not the sex of an animal affects the incidence of disease or the severity of EAE can be complicated, as these parameters also vary with the species, strain and age of animal and the antigens and adjuvants that are used to induce disease (46–53). In addition, in many EAE studies, animals of only one sex have been tested (most often females, which are often favored as they tend to be less aggressive to each other when housed in groups and also because of the higher prevalence of MS in females), or else the sex of the animals is sometimes not reported in papers.

### Actively-induced EAE

In older studies using outbred animals (e.g. non-human primates and guinea pigs), where both sexes have been tested, males and females generally appear to be equally susceptible to development of EAE (54–57). Only some rabbit strains are able to develop EAE, but, in those susceptible strains, the incidence of disease is reported to be similar in males and females (36).

In inbred rat and mouse strains, however, there are many differences reported between males and females (**Table 1** – details of variables such as strain, age, antigen etc are given when this information is available). For example, adult (8-10 week old) Lewis rats of both sexes can develop EAE, but in males disease is generally monophasic, whereas females of the same age often develop relapses of disease (58–60); however, juvenile (6-week-old) male Lewis rats develop more severe relapsing EAE than juvenile females. In contrast, in adult DA rats, both males and females develop relapsing EAE with similar clinical severity; however, by histology, males show more severe inflammation, whereas females have more astrocyte activation (47). To round out the spectrum of responses, in the PVG strain of rats, males are much more susceptible than females to development of EAE and disease severity is greater in the males (63).

**Table 1:** Effects of biological sex on incidence and severity of actively-induced EAE in rodents.

Species/Strain	EAE-inducing antigen	Differences/Similarities between males and females	References
Adult Lewis rats	gp SCH	Incidence, day of onset, and disease severity same in both. In Keith, 1978, 45% of females develop relapsing disease, whereas disease course is only acute in males.	(58, 59)
Adult Lewis rats	Bovine PLP	Incidence, day of onset and disease severity same in both. Relapses in 25% of female rats.	(60)
Juvenile Lewis rats	Human MBP	6 week old males have a higher incidence and develop more severe disease than 6 week old females, and all males that develop EAE have a relapsing course.	(61)
Dark Agouti (DA) rats	Rat rMOG_1-125_	Both males and females develop chronic EAE. No differences in incidence or disease severity	(21, 62)
DA rats	gp SCH	Both males and females develop relapsing EAE. Males have higher inflammation score, but females have more astrocyte activation. Demyelination similar in both.	(47)
PVG rats	gp MBP	Females resistant to EAE (30% develop very mild disease). Males highly susceptible to EAE (83% develop severe disease).	(63)
C57BL/6 mice (H-2^b^)	MOG_35-55_	Incidence the same in males and females. Males have slightly earlier day of onset but lower maximum clinical score. Disease course is chronic in both. Young males develop less severe EAE than middle-aged males.	(48, 49) (64)
NZW/LAC mice (H-2^d^)	gp MBP	Higher incidence in females (85%) vs males (25%), but males that develop EAE have higher maximum EAE score than females. Disease course chronic in both. Mice were used at 3-4 months of age.	(49)
NOD/Lt mice (H-2^g7^)	MOG_35-55_	No differences between males and females in incidence (~90%), day of onset of disease, clinical scores or disease course (relapsing)	(49)
SJL/J mice (H-2^s^)	Mouse SCH	Incidence in 6 week old mice was significantly lower in males (5%) than females (67%), but day of onset and disease score in mice that developed EAE was similar. However, 12 weak old males had a higher incidence of EAE (57%).	(65)
SJL/J mice (H-2^s^)	PLP_139-151_	Increased incidence in females vs males (97 vs 80% in 5-6 week old mice, and 90 vs 30% in 6-8 week old mice), slightly increased clinical score in females vs males at both ages. Relapses in ~70% of females. No relapses in males.	(46, 49)
SJL/J mice (H-2^s^)	MOG_92-106_	Incidence slightly higher in females (100%) vs males (80%). Day of onset of disease earlier in females than males. Clinical scores higher in females. Both males and females developed relapsing EAE.	(49)
SJL/J mice (H-2^s^)	ACA_83-95_	No sex differences when EAE actively induced.	(66)
A.SW mice (H-2^s^)	MOG_92-106_	No differences between males and females in incidence (80%), day of onset of disease, clinical scores or disease course (chronic), but higher mortality in females than males (40% vs 20%)	(49)
(B10.S x SJL) F1 mice (H-2^s^)	Mouse SCH	Females more likely to develop EAE. Age dependent.	(51)
PL/J mice (H-2^u^)	MOG_92-106_	Incidence 100% in both males and females. Similar day of disease onset and severity of clinical scores. Low mortality in males, none in females	(49)
B10.PL mice (H-2^u^)	gp MBP	No differences between males and females in incidence (100%), day of onset of disease, clinical scores or disease course (chronic), but higher mortality in males than females (24% vs 4%)	(49)

ACA, Acanthamoeba castellani (peptide ACA_83-95_ cross-reacts with PLP_139-151_); gp, guinea pig; MBP, myelin basic protein; MOG, myelin/oligodendrocyte glycoprotein; PLP, myelin proteolipid protein; SCH, spinal cord homogenate.

Similarly, in mice, there are strain-specific relationships in the effects of sex on EAE, but these can be quite complex (**Table 1**). For example, in the SJL mouse strain with EAE induced with a variety of antigens (CNS tissue homogenates, PLP or MBP), females have generally been found to be significantly more susceptible to EAE than males (>80% vs <30%, respectively), have an earlier time of onset of disease, and have higher clinical scores compared to males (49, 52, 67). However, this female predominance in SJL mice is not seen when EAE is induced with MOG_92-106_ peptide (49). In addition, the observed sex differences can be age dependent: 6-8 week adult SJL males are resistant to induction of PLP-induced EAE, however, the risk for development of EAE in males increases by 4% for each increasing week of age; in contrast, disease severity does not change in females with ageing (51, 68, 69).

It does not appear that the major histocompatibility complex (called the H-2 complex in mice) allele carried by the mice has a major role in the effects of biological sex on development of EAE, as other strains of mice carrying the H-2^s^ allele (same as SJL) either show no significant differences in disease incidence, clinical scores, or disease course between males and females (e.g. A.SW strain), or else both sexes are resistant to development of EAE (e.g. B10.S strain). The only other mouse strains in which females are reported to be significantly more susceptible to actively-induced EAE compared to males are (B10.S x SJL)F1 mice (H-2^s^) and the NZW/LAC strain (H-2^d^), although, in the latter strain, males that show clinical signs have more severe disease than females (49). In mouse strains such as C57BL/6 (H-2^b^), NOD (H-2^g7^), and PL/J and B10.PL (both H-2^u^), the incidence of EAE in male and female mice does not differ significantly between the sexes (48–50, 70), but male PL/J and B10.PL mice show increased EAE-related mortality compared to females (49). Interestingly, a recent report suggests that a female bias in disease severity becomes more apparent in C57BL/6 mice when lower amounts of pertussis toxin are used during the induction phase of EAE, suggesting that a sex difference in the response to pertussis toxin could confound other underlying sexual dimorphism in this strain (53).

### Spontaneous EAE in transgenic mice

Several “spontaneous” EAE models have also been developed, in which there is transgenic expression of immunoglobulin heavy (IgH) chains and/or T cell receptors (TCR) specific for peptides of MOG on either C57BL/6, NOD, or SJL backgrounds (20, 71–73). Some of these models exhibit sexual dimorphism (**Table 2**). Double transgenic (i.e. IgH X TCR) mice on a C57BL/6 background show no sex differences (72), however all the other models show a higher incidence of spontaneous disease in females. In spontaneous models on an SJL background, female mice are more likely to develop a relapsing-remitting type of disease, whereas males mostly develop progressive disease from the outset (20, 73).

**Table 2:** Effects of biological sex on incidence and severity of spontaneous EAE mouse models.

Details of model	Differences/Similarities between males and females	References
Double transgenic 2D2 (MOG _35–55_–specific TCR) x IgH^MOG^ mice on C57BL/6 background (H-2^b^)	No sex differences.	(72)
Double transgenic 1C6 (MOG _35–55_–specific TCR) x IgH^MOG^ mice on NOD background (H-2^g7^)	Incidence of spontaneous EAE higher in females (~80%) than males (45%), but no differences in day of onset or disease severity.	(71)
TCR^1640^ mice on SJL/J background (H-2^s^)	99% of T cells in the TCR^1640^ mice are specific for MOG_92-106._ Higher incidence of disease in female (80%) vs male (60%) mice. Females much more likely to develop a relapsing-remitting type of EAE, whereas males mostly develop progressive disease from the outset.	(20, 73)
Double transgenic TCR^1640^ x IgH^MOG^ mice on SJL/J background (H-2^s^)	Incidence of spontaneous disease >80% in females vs 40% in males within 120 days. Females much more likely to develop a relapsing-remitting type of EAE, whereas males mostly develop progressive disease from the outset.	(20)

### Adoptive transfer EAE

Adoptive transfer studies have been done to try to determine if the sex of the donor of the encephalitogenic T cells affects the susceptibility to development of EAE, or if is the sex of the recipient that determines the outcome. These studies have yielded conflicting results (**Table 3**). A confounder in the interpretation of these studies is that each has used a different mouse strain and/or antigen. The different studies variously suggest that :i) the sex of the recipient determines the differences (52: ii) that disease severity is related to the sex of the donor cells, with female cells giving greater disease, and suggesting that the sex differences play a greater role during the induction phase of the disease (74); or iii) that cells from male donors induce more severe disease, irrespective of whether they are transferred into male or female recipients (75). *In vitro* conditions under which the cells to be transferred are prepared may also affect the outcome, as differences in findings from adoptive transfer studies do not always reflect the sex bias seen in actively induced EAE (66).

**Table 3:** Effects of biological sex in adoptive transfer EAE.

Donor cells	Recipients	Effect	Interpretation	References
MBP reactive T cells from female or male SJL mice	SJL mice of both sexes	Disease more severe in females	Sex of recipient determines the difference	(52)
				(52)
PLP_139-151_ reactive cells from male and female SJL mice	Female SJL mice	Disease more severe after transfer of female cells	Severity of EAE is related to the sex of the donor cells	(74)
MOG_35-55_ reactive Th17 cells from 1C6 TCR transgenic mice (NOD background) of both sexes	Female or male NOD.*scid* mice	Male Th17 cells caused greater disease than female cells in both female and male recipients	Sex of the donor determines the difference. Male Th17 cells have a greater intrinsic pathogenic capacity than female Th17 cells.	(75)
ACA_83-95_ reactive T cells from female or male SJL mice	SJL mice of both sexes	No difference in disease between males and females following transfer of female cells. No disease in female recipients and low incidence and severity of disease in males following transfer of male cells.	Disease-inducing ability of T cells is influenced by the sex of both the donor and recipient.	(66)
MOG_92-106_-specific T cells from female or male TCR^1640^ TCR transgenic mice (on SJL/J background)	SJL/J mice of both sexes	Relapsing-remitting disease course with increased clustering of Foxp3^+^ cells during remission in both males and females following transfer of female cells. Progressive disease course without recovery and more Th1 than Th17 lymphocytes in CNS during chronic phase in both males and females following transfer of male cells.	Disease course is dependent on the sex of the donor.	(73)

### In summary

Overall, we can conclude that some mouse strains show a female bias in disease, but this may either not occur in other strains, or be masked by sex-related effects of the antigens or adjuvants used. In most studies, females appear more likely to develop a relapsing-remitting disease course, whereas males are more likely to develop a chronic progressive disease course with greater inflammation than seen in females, which is somewhat reminiscent of what occurs in humans with MS.

## Effects of biological sex on various parameters relevant to the pathophysiology of EAE

A summary of some of the cells and molecular pathways involved in sex differences in the pathophysiology of EAE are given in **Tables 4** and **5** and are discussed in more detail below.

**Table 4:** Cells that are implicated in sex differences in EAE.

Cells	Model	Effect	References
Immune cells			
Th1 cells	PLP_139-151_-induced EAE in SJL mice	Lymph node and spleen cells from female mice produce more IFN-γ and are more pathogenic than male cells.	(76)
Th17 cells	Adoptive transfer 1C6 TCR transgenic mice into NOD.*scid* mice	Male derived Th17 cells are more pathogenic	(75)
ILC2 cells	PLP_139-151_-induced EAE in SJL mice and SJL-*Kit^W/W-v^* mutant mice	Mature ILC2s accumulate in draining lymph nodes and CNS of male mice (EAE resistant), but not in female mice. Male SJL-*Kit^W/W-v^* mutant mice, which show deficiency of mature ILC2s, develop more severe EAE than their wild type male counterparts.	(77)
Th2 cells	PLP_139-151_-induced EAE in SJL mice	Males selectively develop a Th2 type of response that prevent relapses	(46)
Macrophages	MBP-induced EAE in PVG rats	Resistance in females linked to NO production by macrophages	(63)
Macrophages	Mouse spinal cord homogenate-induced EAE in SJL mice	Resistance of young male mice to induction of EAE due to lack of a specific macrophage population.	(65)
Macrophages/monocytes	Adoptive transfer of MOG_35-55_-specific T cells into SV.129 (H-2^b^) mice	Male mice are resistant to EAE through sex-specific expression of PPARα and PPARγ by macrophages/monocytes	(78)
Mast cells	PLP_139-151_- induced EAE in SJL mice	Androgen-responsive mast cells in males produce more IL-33 during EAE induction, which activates ILC2 to promote and sustain a Th2 type of response and prevent EAE.	(79)
Treg cells	PLP_139-151_-induced EAE in B10.S mice	Depletion of Treg cells enhances disease susceptibility in males	(80)
Treg cells	MOG_35-55_-induced EAE in C57BL/6 mice	Reduced number of Treg cells in older males leads to more severe disease	(64)
**CNS resident cells**			
Astrocytes	MOG_35-55_-induced EAE in C57BL/6 mice expressing the RNA regulator HuR	Less severe disease in female HuR^+^ mice compared to wildtype females. Effect due to gonadal hormones.	(81)
Astrocytes	MOG_35-55_-induced EAE in C57BL/6 mice	Expression of complement by astrocytes in optic nerve is increased in females vs males and correlates with worse disease	(82)
Oligodendrocytes	gpSCH-induced EAE in DA rats	Healthy males show higher expression of myelin markers MBP and PDGFα receptor than females, but females show greater resistance than males to downregulation of these markers during EAE.	(47)
Microglia	MOG_35-55_-induced EAE in microglial-depleted C57BL/6 mice	Re-engraftment of microglia exacerbates disease in females but not males	(83)
Microglia	MBP-specific T cells from SJL mice of either sex incubated with microglia	T cells from females and castrated males, but not intact males, induced iNOS in co-cultured microglia	(84)

**Table 5:** Molecules and pathways implicated in sex differences in EAE.

Molecule/Pathway	Model	Effect	References
ApoE	MOG_35-55_-induced EAE in C57BL/6 wildtype or apoE-deficient mice	Apo E deficiency led to less severe disease in males but more severe EAE in females compared to wildtype mice.	(85)
Axonal injury markers	MOG_35-55_-induced EAE in C57BL/6 mice	Greater axonal amyloid precursor protein (APP) and non-phosphorylated neurofilament heavy chain expression and greater dendritic branching in males vs females	(86)
CD152 (CTLA-4)	PLP_139-151_-immunized SJL mice	Activated Th cells from females have reduced expression of CD152 (CTLA4) compared to males	(87)
Complement	MOG_35-55_-induced EAE in C57BL/6 mice	Greater complement expression in optic nerves of females; associated with greater axonal loss in females	(82)
Cystatin C (*Cst3*)	MOG_35-55_-induced EAE in *Cst3* ^null^ C57BL/6 mice	No effects in males. Females showed reduced severity of clinical signs of EAE, which was associated with lower expression of molecules involved in T cell activation.	(88)
HuR	MOG_35-55_-induced EAE in C57BL/6 mice expressing the RNA regulator HuR	Less severe disease in female HuR^+^ mice compared to wildtype females. Effect due to gonadal hormones.	(81)
IFN-γ	PLP_139-151_-induced EAE in SJL mice	Lymph node and spleen cells from female mice produce more IFN-γ and are more pathogenic than male cells.	(76)
IL-13	PLP_139-151_-induced EAE in SJL-cKit mutant mice	Males have increased susceptibility, due to decrease in IL-13 production by ILC2 cells and switch from Th2 to Th17 response. Females more resistant to EAE development.	(77)
IL-13	MOG_35-55_-induced EAE in IL13C57BL/6 mice	Knockout or IL-13 reduced incidence and severity of EAE in females but not males.	(50)
IL-17	PLP_139-151_-induced EAE in SJL mice and MOG_35-55_-induced EAE in C57BL/6 mice	Male mice produce more IL-17 than females	(75, 76)
IL-33	PLP_139-151_- induced EAE in SJL mice	IL-33 selectively induced in androgen-responsive mast cells in males, which activates ILC2 to promote and sustain a Th2 type of response and prevent EAE. Treatment of female mice with IL-33 reduces disease in females. Treatment of male mice with anti-IL-33 antibody made them more susceptible to EAE.	(79)
MBP	gpSCH-induced EAE in DA rats	Sex difference in MBP expression during remyelination	(47)
iNOS/NO	MBP-induced EAE in PVG rats	Females resist EAE with increased NO production by macrophages	(63)
iNOS/NO	Passive transfer of MBP-specific T cells in SJL mice	T cells from female donors induced more severe disease and higher expression of iNOS and NO in female vs male recipients. T cells from male donors induced only mild disease with no iNOS or NO.	(89)
Oxidative stress markers	Rat SCH-induced EAE in DA rats	Males had increased markers of oxidative stress	(90)
p38MAP kinase	MOG_35-55_-induced EAE in C57BL/6 mice	Inhibition reduced EAE in females, not males	(91)
PDGF alpha receptor	gpSCH-induced EAE in DA rats	Sex differences in expression during remyelination	(47)
S1P receptor 2	PLP_139-151_-induced EAE in SJL mice and MOG_35-55_-induced EAE in C57BL/6 mice	Increased expression in SJL females vs males, leading to enhanced vascular permeability in CNS tissue of females but not males. Equal expression in both sexes in C57BL/6 mice with MOG-induced EAE	(92)
TLR7	Bone marrow chimeric SJL-FCG mice with PLP-EAE	Presence of XY sex complement in the CNS leads to more severe EAE, due to increased expression of TLR7 in the CNS	(93)
Vitamin D metabolism	gpMBP-induced EAE in B10.PL(73NS)/Sn mice	Vitamin D3 protective in female but not male mice; females have enhanced ability to metabolize Vitamin D3 to active hormone	(94)

### Effects on the adaptive immune response

When considering the effects of biological sex on the outcome of EAE, it is important to remember that the sex of an animal impacts the makeup of the immune cells and organs under physiologically normal conditions, as well as when they are pathologically challenged. This is true of humans, as well as experimental animals, where (for example) CD4^+^ cells from female healthy volunteers show a much higher levels of production of IFNγ following T cell activation with anti-CD3/anti-CD28 than do cells from males, whereas IL-17 production is higher in cells from males (76). In humans, elevated Th17 responses have been linked to male sex in some autoimmune diseases (95).

In mice, biological sex affects the numbers and activity of Th1, Th17 and Th2 T cells, regulatory T cells (Treg), and antibody levels (96, 97). However, these differences are not necessarily seen to an equal degree in all immune organs in an individual (e.g. there can be variation between the sex-related differences seen in the spleen and those seen in other secondary lymphoid organs) and they can occur in a mouse strain-specific manner (96).

Effects of biological sex on adaptive immune responses in EAE are summarized in **Table 4**. Neuroantigen-specific CD4^+^ Th1 and Th17 cells are the main cells responsible for the induction of EAE. Although many papers state that Th17 cells are critical for disease development, there are quite a number showing that, at least in both the models of MOG-induced EAE in C57BL/6 mice and PLP-induced EAE in SJL mice, Th1 cells, Th17 cells, and cells producing both IFNγ and IL-17 appear to be equally able to induce EAE (76, 98–101). However, there do appear to be differences in the site of induction of antigen-specific T cells in male and female mice. For example, in PLP-induced EAE in SJL mice, lymph nodes cells from female mice produce copious amounts of both IFN-γ and IL-17 in response to a secondary challenge with the autoantigen, whereas lymph node cells from male mice produce neither of these. In contrast, spleen cells from the same mice produce predominantly IFN-γ if they are from females, but IL-17 if they are from males (76). These observations have been interpreted as providing an explanation for the ability (or not) of male mice to develop EAE, based on how much IL-17 they can produce.

However, the interpretation of these results may be more complicated. A recent study found (using adoptive transfer of MOG-specific transgenic Th17 cells from males and females into sex-matched NOD.*scid* recipients) that male Th17 cells are more encephalitogenic than female Th17 cells, but only if they rapidly switch (once they are transferred into the recipient) from producing IL-17 to producing large amounts of IFN-γ- cells from males that produce some IFN-γ, but also retain IL-17 production, as is seen in females, are less encephalitogenic (75). Relative levels of IFN-γ and IL-17 therefore appear to play different roles in the pathogenesis of EAE in males vs females, but much work remains to determine the exact relationship between these measures and the susceptibility of different strains of mice to EAE and how (or whether) this relates to humans with MS, in whom similar mixtures of myelin antigen-specific Th1, Th17 and mixed phenotype cells have also been reported (102).

There are several molecules that are of potential interest in these effects, including PPARγ, one of the central regulatory molecules in determining T cell fate, which selectively inhibits Th17 cell differentiation only in male cells and which modulates Th1, Th2 and Th17 differentiation in female T cells, based on the levels of estrogen (103), and Jarid1c, an X-linked immune regulator that represses severity of Th17-mediated EAE in males (75).

There also appear to be sex-based differences in the action of regulatory types of T cells in EAE. In PLP-induced EAE in SJL mice, male mice develop IL-10 producing cells that prevent relapses of disease (46) and this appears to be due to direct effects of testosterone on CD4^+^ T cells (104), whereas females have increased T cell proliferation and reduced expression of the inhibitory molecule CD152 (CTLA-4) compared to males (87). In B10.S mice, which are normally resistant to PLP-induced EAE, male mice show greater susceptibility to disease after depletion of Treg cells, but depletion of Treg cells has no effect on susceptibility of female mice (80). In MOG-induced EAE in C57BL/6 mice, where there are no sex differences in incidence of disease, there appear to be underlying sex differences in pathophysiology, as male mice have both increased inflammatory processes and increased regulatory processes, which cancel out, so that disease is similar to that in females (97). Another study using the same model found that young male mice develop less severe EAE than middle aged male mice, due to reduced numbers of CD4^+^CD25^+^ Treg cells in the middle-aged vs the younger males (64).

### Effects on the innate immune response

As with cells of the adaptive immune response, there are differences between numbers and location of dendritic cells (DC), macrophages, natural killer (NK) cells and other innate cells in male and female mice (96, 97). In actively-induced EAE studies, an important consideration when looking at innate immune responses is the effects of the adjuvants (typically complete Freund’s adjuvant and pertussis toxin) used in the induction of disease, as a recent study (97) found that there were sex differences in the induction of key innate inflammatory factors by these adjuvants alone.

Several sex-related differences in macrophages have been reported in EAE (**Table 4**). Resistance of male SJL mice to induction of EAE is reportedly dependent on a subpopulation of macrophages (Mac-1^+^, Mac-2^-^, Mac-3^+^) that play a regulatory role in the induction of encephalitogenic T cells (65). Additionally, several rodent models have associated higher levels of inducible nitric oxide synthase (iNOS) and nitric oxide (NO) production by macrophages with sexual dimorphism in EAE; however, in PVG rats, this is linked to resistance to EAE in females (63), whereas in SJL mice it is linked to increased severity of EAE in females (89). Other molecules produced or expressed by macrophages or monocytes that have been implicated in the sexual dimorphism seen in EAE include PPARα and PPARγ (78), and several Th2 cytokines, including IL-33. In addition, apolipoprotein E (apoE), which is produced by macrophages and DCs, has been reported to enhance anti-inflammatory macrophage phenotypes and downregulate Th1 and Th17 responses (105, 106). It is therefore not surprising to find that apoE deficiency in C57BL/6 mice with MOG-induced EAE leads to more severe EAE in females; however, it is surprising to find less severe EAE in apoE-deficient males (85). The mechanisms underlying these varying effects of apoE deficiency in males and females remain to be elucidated. Finally, inhibition of p38 MAPK activity in C57BL/6 mice with MOG-induced EAE ameliorates EAE in female but not male mice, and this is due specifically to affects in myeloid cells of the females, which have a greater dependence on p38 signaling for expression of proinflammatory genes than do male myeloid cells (91).

Other IL-13- and IL-33-expressing cells, including mast cells and innate lymphoid cell type 2 (ILC2) cells, are also of relevance for sex-related differences in EAE. In PLP-induced EAE in SJL mice, androgen receptor-expressing mast cells from males produce higher levels of IL-33 in response to testosterone and other inflammatory activators (79). IL-33 then activates ILC2 cells to promote and sustain a Th2 type of response (via production of IL-13) and prevent EAE. Treatment of male mice with anti-IL-33 antibody makes them more susceptible to EAE, whereas treatment of females with IL-33 leads to a reduction in EAE severity (79). Conversely, decreased accumulation of IL-13-secreting ILC2 cells in the brains of male, but not female, SJL–c-Kit mutant mice leads to increased susceptibility to development of EAE in the male mice, but resistance to the development of EAE in females (77), *via* a switch from a Th2- to a Th17-dominated T cell response that is regulated by the presence or absence of mature ILC2 cells in the draining lymph nodes and the CNS. In C57BL/6 mice, knockout of IL-13 reduces the incidence and severity of MOG-induced EAE in females but not in males (50).

Within the CNS, microglia and astrocytes are the main cell types thought to be involved in innate responses. They differ regionally and numerically between male and female mice (107–111) and there is sexual dimorphism in their responses to CNS assault in adult mice (107, 112). For microglia, these can include differences in inflammatory sensitivity and reactivity, their ability to affect cellular repair, and in their response to oxidative stress (113, 114). Studies in which microglia have been depleted from brains of mice using the CSF1R inhibitor, PLX5622, result in adverse sex-specific behavioral effects, with females developing long-term hyperactivity and anxiolytic-like behaviour (115). Astrocytes display sex differences in uptake of glutamate and their responses to fatty acids, and are known to express the estrogen receptor alpha (ER-α) (116–118). There are also sex differences in mitochondrial bioenergetics of astrocytes, but not microglia (119).

In EAE studies, some sex-specific effects on or by these CNS cell types have also been reported (**Table 4**). In microglia, for example, iNOS can be induced by MBP-primed T cells from female and castrated male SJL mice, but not from intact males, and this is dependent on activation of a CCAAT-enhancer-binding protein (C/EBPβ) in the microglia (84). In MOG-induced EAE in microglial-depleted C57BL/6 mice, re-engraftment of microglia exacerbates disease in females but not males (83). Astrocytes are also involved in the pathology of EAE (120) and sex differences in the astrocyte response during EAE have been described. For example, there is expression of complement by astrocytes in optic nerves of C57BL/6 mice with MOG-induced EAE, and this is greater in females than males and correlates with worse retinal ganglion cell and axonal loss in females (82). Similarly, transgenic expression in astrocytes of C57BL/6 mice of the RNA regulator HuR, which plays a major role in regulating production of cytokine and growth factors, results in attenuation of MOG-induced EAE in females, but not males (81). Furthermore, this effect can be reversed by ovariectomy.

One molecule that is widely expressed in both peripheral and CNS innate immune cells, and which has previously been implicated in both MS and EAE, is cystatin C. However, cystatin C appears to have different functions in immune and CNS cells, and there has been much debate on whether it plays a protective or pathogenic role in MS and EAE. In MOG-induced EAE in C57BL/6 mice, deficiency of *Cst3*, the gene encoding cystatin C, leads to improvement of EAE in females, but has no effect in males. Furthermore, the sex-dependent effects of cystatin C are negated by ovariectomy of females, and are revealed by treatment of males with female hormones (88).

### Effects on other cells and systems, and on responses to therapy

Immunometabolism, the metabolic reprogramming of anaerobic glycolysis, oxidative phosphorylation and metabolite synthesis that occurs when immune cells are activated, is an important regulator of immune responses and immune homeostasis. Immunometabolic dysfunction has been linked to an inflammatory phenotype of immune cells and has been implicated in development of autoimmune diseases (121), including EAE (122). There is an interaction of immunometabolism with sex, especially through the actions of reproductive hormones (123). Male DA rats with EAE have more severe disease and greater oxidative stress than females (90), consistent with studies that show sex differences in levels of pro-oxidant and anti-oxidant enzymes (124, 125). It has also been found that high sodium (126), high fat (127), or high carbohydrate (128) diets can all exacerbate EAE. In SJL mice fed a high sodium diet from prior to induction of PLP-induced EAE, there is an increase in disease severity in female mice, whereas male mice are not affected (129). In contrast, compared to mice fed a normal diet, both male and female C57BL/6 mice with MOG-induced EAE and fed a high sodium diet show some increase in disease severity, particularly during the peak of the acute disease (129). *In vitro* studies have suggested that the effects of high levels of salt appear to be due to stabilization of the Th17 cell phenotype, *via* the activation of several pathways, including p38 MAPK, nuclear factor of activated T cells 5 (NFAT5), and the salt-sensing serum glucocorticoid kinase 1 (SGK1) (126, 130). However, a high salt diet has also been reported to elevate serum sodium, leading to increased blood-brain barrier permeability and more severe brain pathology (129). Additionally, it cannot be ruled out of these studies that different effects of high salt diets in mice of different sexes may relate to changes to the gut microbiome. The gut microbiome appears to play an important role in both EAE and MS (131). Sex hormones influence the composition of the gut microbiota (132) and its interaction with the brain (“the gut-brain axis”) (133). In MOG-induced EAE in C57BL/6 mice, protection due to estrogen is associated with changes in the gut microbiota and the gut lymphoid tissue (134). In the same model, oral administration of 2.6% alcohol for 3 weeks prior to EAE induction is protective in both sexes, but the protective effect is greater in males, and also appears to be mediated though changes in the gut microbiota (135).

Several therapeutic interventions also show sex-specific effects. Supplementation with Vitamin D3 protects against EAE in female but not in male mice of several strains (94, 136), and this effect appears to be due to sex differences in vitamin D3 metabolism, with females having an enhanced ability to metabolize vitamin D3 to the active hormone 1,25-(OH)_2_D3 (94). Ovariectomy abrogates this protective effect (94). Other therapeutic approaches show more of a male-specific effect, e.g. oral tolerance can be induced in male, but not female, B10.PL mice fed with an altered peptide ligand of MBP (137).

In several autoimmune diseases it is recognized that the severity of disease can be influenced by the resistance of the target tissue to damage (138, 139). In EAE and MS, target organ resistance could involve variability in the processes that lead to damage to the myelin-producing oligodendrocytes and neurons, and in the processes of repair. Since myelin is the target of immune attack in EAE and MS, several studies have investigated the effects of biological sex on oligodendrocytes. These studies have shown that there is sexual dimorphism in oligodendrocytes’ capacity for myelination and their responses to stress (140, 141). There are reports of sex differences in expression of mRNA for MBP and PDGF alpha receptor (markers of myelination) in DA rats with EAE (47), and markers of axonal injury and dendritic aborisation are greater in male than female C57BL/6 mice with MOG-induced EAE (86). However, it is not clear at present if these findings merely reflect the strength of the immune response in the animals of different sexes in these models or if they truly reflect sex differences in target organ resistance in EAE. Adoptive transfer studies of male and female cells (not only of peripheral immune cells into naïve recipients, but also of microglia into microglia-depleted recipients) could help to answer this question. This is a topic that will likely be difficult to decipher, but one that may prove very useful in ultimately answering some important questions related to the differential prognosis of males and females with MS.

Other molecules that are expressed in the CNS can also influence sex differences in other pathophysiological mechanisms in EAE. For example, sphingosine-1-phosphate receptor 2 (S1PR2) is a molecule involved in regulation of the integrity of the blood-brain barrier (BBB). Naïve female SJL mice have significantly elevated levels of S1PR2 compared to SJL males and to C57BL/6 mice of both sexes (92). Induction of PLP-induced EAE in the SJL mice further increases expression of S1PR2 and causes increased vascular permeability in CNS tissue from females, but not males. Furthermore, S1PR2-deficient mice showed decreased EAE severity compared with wild-type controls (92). Interestingly, however, adoptive transfer of CD4*^+^* cells from TCR1640 transgenic mice (MOG**_92-106_**-specific cells on SJL background) into naïve male or female SJL recipients show no sex differences in BBB integrity (73).

It may even be that the sex of the experimenter can affect outcomes. Whilst this has not been systematically investigated in EAE, as far as we are aware, there are several reports that rodents of both sexes are more stressed when the person doing the experiments on them is male, and that this can lead to differences in behavioral assays of pain and stress (142, 143). This could potentially add another layer of complexity to investigation of the effects of biological sex on the pathophysiology of EAE.

### In summary

Overall, biological sex can affect many of the elements of immune function and CNS biology that are required for development of EAE. Sex differences could occur during the induction of disease, where there could be sex differences in response to adjuvant and in antigen presentation or in the effector stage of disease when there could be sex differences in the pathological processes in the CNS. To clarify this, appropriate controls (naïve and adjuvant only) of both sexes need to be included in all experiments looking at mechanisms underlying sex differences in EAE. Much more work remains to understand the basic biological differences between the sexes, both in mice and humans.

## Role of sex chromosomes and sex hormones in sexual dimorphism in EAE

### Sex chromosomes

The sex chromosomes, X and Y, are the only chromosomes in the body that are represented differently in females and males, and they vary greatly (144). Thus, effects of sex could be due differences in the sex chromosomes and/or sex-related differences in gene expression.

The Y chromosome contains only 50 to 60 unique protein-coding genes that encode primarily for molecules required for gonadal formation and fertility, including the *Sry* gene, which is necessary for the development of male gonads (145) and the *Azf* (azoospermia factor) locus that has a role in organs outside the testis, including the brain (146), and in inflammation (147, 148). Variable amounts of gene replication occur on the Y chromosome from different species, so that the Y chromosomes from humans and mice are quite different (149, 150). Even within mouse strains differences can be observed: e.g. some strains have a Y chromosome gene known as *Yaa* (y accelerated autoimmunity) which has resulted from a translocation of the gene encoding Toll-like receptor 7 (*Tlr7*) from the X chromosome onto the Y chromosome. Yaa can modulate a mouse’s potential to develop autoimmunity *via* epistatic interactions with other autoimmunity-related genes (151) (152).

In contrast to the Y chromosome, the X chromosome contains over 900 genes, and is highly conserved across species. Genes on the human X chromosome include genes for reproduction and a disproportionately large number that code for brain function, such as the *BEX* family (brain expressed X-linked genes), *NF2* (neurofibromin 2), *GDNF* (glial cell derived neurotrophic factor) and *PLP1* (myelin proteolipid protein, the most abundant protein in CNS myelin, an autoantigen for EAE, and a target of immune attack in MS (153, 154)). The X chromosome also contains genes for the testis that are subject to selection pressure because they confer advantage in males (155). There are also many genes of relevance to immune function and susceptibility for autoimmunity located on the X chromosome, including those encoding IL2-Rγ, IL13-Rα, CXCR3, CD40 ligand, TLR7 and TLR8, TIMP1 and Bruton’s tyrosine kinase (BTK).

In males, the X chromosome is always of maternal origin. In females, one X chromosome is randomly inactivated in each cell of an early-stage embryo, so that the embryo is a mosaic of cells with either an active paternal X chromosome or an active maternal X chromosome. All descendants of that cell will have the same active X chromosome, and most females have an approximately even spread of paternally- and maternally-derived active X chromosomes (156). However, this inactivation is not always complete and can differ in different tissues and cell types from a single individual (157), potentially leading to X dosage effects, with higher expression of X-linked genes in females compared to males. DNA methylation plays an important role in X chromosome inactivation and recent studies have reported that maternally derived X chromosomes have less DNA methylation than paternally-derived X chromosomes, and that therefore there is greater expression of X-linked genes in male cells (158). These factors could have implication for sex differences in autoimmunity.

The development of a mouse model known as the “4 core genotypes” (FCG) model, in which the sex chromosome complement is unrelated to the animal’s gonadal sex (159), has allowed studies of the effects of the sex chromosome complement on susceptibility to EAE. Early studies compared gonadally-intact FCG mice on a SJL background, (SJL-FCG mice) with FCG mice in which the gonads were removed. These studies show that the presence of XY (male) complement of sex chromosomes leads to greater immune responses to MBP than XX chromosomes, whereas the presence of male sex gonads (and therefore sex hormones) inhibits immune responses to MBP (160). In that same model, when gonads are removed, mice carrying the XX genotype are more susceptible to PLP-induced EAE than are mice carrying a XY genotype, thus demonstrating an autoimmune-enhancing effect of the XX genotype independent from hormonal influences (161). Whether this outcome could be due to dosage effects of the target antigen, PLP, which is encoded on the X chromosome, was not investigated. Bone marrow chimeric SJL-FCG mice, in which the mice have either a XX or XY sex complement in the CNS and an immune system with a common sex chromosomal type, demonstrate that the presence of an XY sex complement in the CNS associates with greater clinical and neuropathological severity of EAE, apparently due to higher expression of TLR7 in the CNS of XY vs XX mice (93). FCG mice on a C57BL/6 background and immunized with MOG show parent of origin effects of DNA methylation of a set of five X chromosome genes in autoantigen-stimulated T cells and B cells, with higher expression in XY than XX genotypes. SJL-FCG mice immunized with PLP also show similar effects, but the X-linked genes affected differ from those seen in the C57BL/6-FCG mice, suggesting that parent of origin effects are not strain specific (158).

The Y chromosome also has an effect on immune cell gene transcription (162) and copy number variation in Y chromosome genes plays a role in paternal transmission of autoimmune disease (163). There is an effect of Y chromosome genes on the incidence and severity of EAE, since studies using progeny of C57BL/6 Y-chromosome substitution strains show that the Y chromosome influences the severity of EAE in both male and female mice (164).

Given the differences between the ability of male SJL and C57BL/6 mice to develop EAE, it is of also of interest to note that the Y chromosome in SJL mice has been inherited from the *Mus musculus domesticus* subspecies, whereas the Y chromosome in C57BL/6 and most other commonly used inbred laboratory mouse strains comes from *Mus musculus musculus* (165). Y chromosome substitution (Y consomic) SJL male mice carrying a *Mus musculus musculus* Y chromosome are more susceptible to EAE than are age-matched SJL males, suggesting that a Y chromosome-linked polymorphism controls the sexual dimorphism to development of EAE seen in SJL mice (68).

It is of interest to note that, as well as genes on the sex chromosomes, there are other sex-specific genetic differences. A study of crosses between EAE-susceptible SJL mice and EAE-resistant B10.S mice identified sex-specific loci that governed susceptibility of SJL mice to relapsing EAE (*eae12*) and monophasic EAE (*eae7* and *eae13*) (69). In a large study of 26 chromosome substitution (consomic) strains, in which mice inherited chromosomes from the EAE-resistant wild-derived PWD mouse strain on a C57BL/6 background, 19 genetic loci on 18 chromosomes were identified that influenced susceptibility to MOG-induced EAE: of these, six were male-specific and four were female specific (166).

### Sex hormones

Males and females also differ in the levels of circulating sex hormones, which change over the lifetime. In females this is especially true during pregnancy, when there are increased levels of many different hormones and also increased levels of mediators related to pregnancy (see section 6 for more on pregnancy-related effects). The effects of physiological levels of the major sex hormones could explain some of the observed effects of biological sex in EAE, as shown through comparisons of intact and gonadectomized mice (167, 168). The effects of sex hormones on EAE could be due to immune modulation and/or neuroprotection, as summarized in **Table 6**. In addition, it appears that there could be bi-directional effects of sex hormones and the pro-inflammatory molecules produced during EAE (178), as SJL mice with PLP-induced EAE have a significantly shorter estrous cycle (normally around 4-6 days (179)) than mice just immunized with adjuvant. The effects of the major sex hormones are summarized below.

**Table 6:** Summary of immune and neuroprotective effects of sex and pregnancy hormones.

Hormone	Immune effects	Neuroprotective effects	References
Estriol	Anti-inflammatory	Neuroprotective, aids remyelination	(168–170)
Progesterone	Anti-inflammatory	Neuroprotective	(171, 172)
Prolactin	Pro-inflammatory (controversial)	Possible role in neurogenesis	(173, 174)
Testosterone	Immune suppressive	Possible role in remyelination	(175–177)

#### Estrogens

Estrogens comprise estrone, estradiol (E2) and estriol (E3). Females have higher levels of estrogens than males, and in females the levels fluctuate with the menstrual cycle and decline after menopause. In pregnancy there are high levels of E2 and estrone in early pregnancy and high levels of E3 in the later stages of pregnancy.

Low doses of estrogen are reported to enhance pro-inflammatory responses, whereas high doses appear to inhibit such responses (180). In humans, higher doses of estrogens can inhibit the T cell-stimulatory capacity of DCs (181), modulate the effects of lipopolysaccharide (LPS) on monocytes (182), and inhibit monocyte adhesion to endothelium (183). Estrogens also influence production of cytokines (including IL-1, IL-6, TNF-α, M-CSF, GM-CSF, and TGF-β) and chemokines and affect T cell trafficking, probably *via* modulation of chemokine receptor expression (184). Furthermore, estrogen can convert CD25^−^ cells to CD25^+^ Treg cells (185) (in humans, pregnancy is accompanied by increased levels of CD25^+^ Treg cells (186)). Thus, enhancement of Th2 responses and induction of Treg cells by high doses of estrogens could ameliorate Th1/Th17-mediated autoimmune diseases and could be one of the mechanisms underlying the beneficial effects of pregnancy on MS. Estrogens, particularly E2, also alter nerve conduction, and there is *in vitro* evidence that estrogen can modulate potassium channels, with effects of nerve conduction (187), and be neuroprotective (168).

Ovariectomy of females has been reported to have variable effects on the outcome of EAE. Ovariectomy of female B10.RIII mice with MBP-induced EAE or of C57BL/6 mice with MOG-induced EAE causes worse disease than is seen in intact female mice, showing that even physiological non-pregnant levels of female sex hormones confer some protection (188) (189). In contrast, in SJL and (B10.S x SJL)F1 mice with PLP-induced EAE, ovariectomy of females does not have a major impact on disease development (76, 190), or even has a slightly protective effect (67). Some of these studies show a role for ERα, one of the three estrogen receptors, which is widely expressed in the immune system and in the nervous system (191, 192), in these effects.

As listed in **Table 7**, there are many studies showing that administration of exogenous estrogen, particularly E2 and E3, suppresses EAE in a variety of models, most likely *via* ERα (encoded by *esr1* in mice) signaling (206). A variety of mechanisms have been reported to underlie the beneficial effects of therapeutic administration of estrogen in different EAE models. These include: i) reduced secretion of matrix metalloproteinase 9 (MMP9) (169), a molecule produced primarily by macrophages and astrocytes in EAE and involved in entry of inflammatory cells into the CNS; ii) enhanced numbers of regulatory immune cells (204, 205), particularly regulatory B cells and microglia (203); iii) induction of a novel population of VLA-4^+^ cells, without lymphocyte markers, that suppress EAE (207); iv) prevention of the changes to the gut microbiota that normally occur with EAE (134); v) reduced astrocyte production of the pro-inflammatory chemokine CCL2 (201); and vi) reduced DC antigen presentation and activation (196). Estrogen is also synergistic with vitamin D3 in protection from EAE in females, although not in males (200).

**Table 7:** Estrogen and EAE.

Type of EAE	Treatment	Effect	References
Chronic relapsing MBP_89-101_-induced-EAE in B10.RIII mice	E2 and E3	Reduced disease severity in both intact and ovariectomized females	(188)
Guinea pig MBP-induced EAE in SJL mice	E3	Protection when given prior to or after induction of EAE *via* increased IL10 production	(193)
MBP_Ac1-11_-induced EAE in Vβ8.2 TCR transgenic B10.PL mice	E2 or E3	Augmented suppression of disease by Vβ8.2 TCR vaccination, through induction of IL10 and TGFβ by MBP-specific Vβ8.2 T cells	(194)
MOG_35-55_-induced EAE in C57BL/6 cytokine (IL-4, IL-10 or IFNγ) knockout mice	E2 (low dose)	Suppressed disease and reduced TNFα production compared to wildtype mice	(195)
MOG_35-55_-induced EAE in C57BL/6 mice	E2	Suppressed disease, reduced DCs in CNS, decreased IFN-γ production by splenic CD11c^+^CD8α^+^ DC	(196)
MBP_Ac1-11_-induced EAE in Vβ8.2 TCR transgenic B10.PL mice	E2	Suppressed disease reduced DCs in CNS	(196)
PLP_139-151_-induced EAE in SJL mice	E2 or oral EE	Both E2 and EE suppressed disease when given at time of EAE induction. EE also reduced relapses of EAE when given at the time of development of clinical signs. Both E2 and EE reduced inflammation with decreased levels of proinflammatory cytokines and MMP9 and increased expression of TGF-β in CNS	(197)
PLP_139-151_-induced EAE in SJL mice or in MBP_Ac1-11_-induced EAE in B10.PL mice	Tamoxifen	Delayed disease onset and reduced cumulative disease scores	(198)
MOG_35-55_-induced EAE in C57BL/6 mice	E3	Suppressed disease and reduced MMP9 levels	(169)
MOG_35-55_-induced EAE in C57BL/6 mice	E2	Suppressed disease, *via* increased expression of PD-1 in Treg compartment, increased frequency of Treg cells, and reduced IL17 production	(199)
Guinea pig MBP-induced EAE in B10.PL mice and MOG_35-55_-induced EAE in C57BL/6 mice	E2	E2 is necessary for suppression mediated by Vitamin D3 in ovariectomized female mice and male mice.	(200)
MOG_35-55_-induced EAE in C57BL/6 mice	E2	Treatment after disease onset suppressed disease, reduced inflammation and astrocyte production of CCL2	(201)
MOG_35-55_-induced EAE in C57BL/6 mice	E2	Suppressed disease, but not in B cell deficient mice	(202)
MOG_35-55_-induced EAE in C57BL/6 mice	E2	Suppressed disease, enhanced regulatory B cells and M2 microglia that are neuroprotective	(203)
MOG_35-55_-induced EAE in intact and OVX females and male C57BL/6 mice	E2	Pretreatment protected all mice from EAE *via* increased regulatory B cells and M2-like macrophages (+ Treg in male and intact females only)	(204)
MOG_35-55_-induced EAE in ovariectomized C57BL/6 mice	E2	Suppressed disease, increased Tregs and regulatory cytokines (IL4, IL10, TGFβ)	(205)
MOG_35-55_-induced EAE in C57BL/6 mice	E2	Protects from EAE and prevents EAE-associated changes in gut microbiota	(134)

E2, estradiol; E3, estriol; EE, ethinyl estradiol (semi-synthetic estrogen compound found in birth control pills).

Although there is a significant amount of evidence that exogenous estrogen can inhibit EAE, clinical application of estrogens in human patients has been slow, due to undesirable side effects of treatment mediated *via* the intracellular ERα. There have therefore been studies of drugs that target other elements of the estrogen pathway, including an agonist, G-1, that selectively activates the putative membrane estrogen receptor, GPR30, without engagement of ERα. G-1 protects C57BL/6 mice against development of MOG-induced EAE *via* enhancement of CD4^+^Foxp3^+^ regulatory T cells (208). Other studies have used selective estrogen receptor modulators such as tamoxifen and raloxifene, which behave as estrogen agonists in some tissues but are either inert or behave like estrogen antagonists in other tissues. These drugs inhibit PLP-induced EAE in SJL mice *via* reduction of antigen presentation by DC and induction of a Th2 bias in myelin-specific T cells (198). They also ameliorate MOG-induced EAE in female C57BL/6 mice by inhibiting NF-κB signaling and expression of CCL20 in reactive astrocytes (209), similar to the effects of E2 (201).

#### Progesterone

Progesterone is present in higher levels in females than males. It is also present in high levels in pregnancy and could contribute to the effects of pregnancy on EAE. Progesterone has immunomodulatory effects that are generally anti-inflammatory, including inhibition of glucocorticoid-mediated thymocyte apoptosis, and reduction of nitric oxide production and expression of toll-like receptors (TLR) by macrophages (210). Progesterone promotes Th2 differentiation *in vitro* and also has neuroprotective effects (211).

In MOG-induced EAE in C57BL/6 mice, progesterone therapy (either prophylactically or therapeutically) leads to a reduction in the severity of disease, increased IL10 production, reduced axonal damage, and increases production of neurosteroids, which may contribute to neuroprotection (212–215). In chronic EAE induced in DA rats, progesterone reduces severity of disease and is neuroprotective (216). Similarly, in Wistar rats with EAE, progesterone therapy slows the rate of deterioration and enhances remyelination (217).

#### Androgens

Androgens control the development and maintenance of masculine characteristics; however, they have other functions, including maintenance of bone mass in both women and men. Androgens are also the precursors of all estrogens. In males, androgens, primarily testosterone, are present in high levels, but these levels decline with increasing age (218). In females, low levels of androgens (particularly the adrenal-derived androgens DHEA and DHEA-S) are present throughout life (219). Testosterone is immunosuppressive and leads to thymic atrophy, while castration of males results in thymic hypertrophy; these effects occur *via* interaction of sex hormones with receptors on thymic stromal cells (220). Testosterone impairs thymocyte proliferation in response to Concanavalin A (175). In men treated with medical castration, there is a reduction in levels of Treg cells, reduced mitogen-induced activation of CD8^+^ T cells, and an increased percentage of NK cells (176), suggesting that testosterone can help preserve immune homeostatis to help prevent autoimmunity.

There is also evidence for effects, some positive and some negative, of testosterone on cells of the nervous system. *In vitro* studies suggest that testosterone can exacerbate cell death in human neurons under oxidative stress conditions, which could be detrimental in MS and EAE, particularly in males (221). However, testosterone is also reported play a role in regeneration of myelin (177); such a mechanism could play a protective role in MS and EAE.

Castration has no effect on disease in C57BL/6 male mice, which are normally susceptible to EAE (222). However, castration of male SJL and (B10.S x SJL)F1 mice, which are normally resistant to development of EAE, increases the incidence and severity of EAE, suggesting that endogenous androgens are protective in males (76, 190). This is also suggested from experiments using male DA rats with SCH-induced EAE, in which levels of testosterone drop during the onset and peak of EAE (223). Androgens have been found to alter the cytokine profile and reduce the pathogenicity of myelin-reactive T cells (224).

Androgen derivatives suppress PLP-induced EAE in female SJL mice and MBP-induced EAE in male B10.PL mice (225). In female SJL mice with MBP-induced EAE, treatment with dihydrotestosterone ameliorates disease and increases IL-10 secretion by T lymphocytes (226). Castration of males removes the sex differences in the induction of oral tolerance to MBP-induced EAE in B10.PL mice (137).

#### Prolactin

Prolactin, a peptide hormone produced mainly by the anterior pituitary gland, has immunomodulatory effects (227), likely due to the presence of the prolactin receptor on T lymphocytes and other cells of the immune system (228). Prolactin promotes the differentiation of thymocytes (229). In general, prolactin appears to enhance autoimmune diseases, and serum levels of prolactin are increased in some autoimmune diseases (230).

Treatment with bromocriptine, which reduces prolactin secretion, ameliorates EAE in female Lewis rats when given up to one week after induction of EAE (231). Since prolactin levels are elevated in the post-partum period, it is possible that the prolactin could induce the relapses and/or worsening of EAE that is often seen during this period.

### In summary

There are effects of both the sex chromosomes and the sex hormones on sexual dimorphism in EAE, but these are not always clear-cut and vary from one EAE model to another. The Y chromosome appears to exert an influence on EAE in both female and male mice through paternal transmission of autoimmune susceptibility. The X chromosome also carries many genes relevant to development of EAE, and X dosage effects may occur. The female sex hormones show fairly subtle effects at physiological levels, but are highly immunosuppressive at higher levels in most models tested. In contrast, male sex hormones appear to have stronger effects at physiological levels.

## Effects of pregnancy on EAE

Pregnancy involves physiological changes in the mother, including elevation of cardiac output, increased basal metabolic rate, increased lipid levels, weight gain and alterations to the immune system (232–237). These changes in maternal physiology during pregnancy and lactation are largely mediated through and reflect the net effects of pregnancy hormones (238). In pregnancy there are changes in the levels of estrone, E2, E3, progesterone, prolactin, early pregnancy factor (EPF) (239), alpha-fetoprotein (240) and leptin (241), and elevated levels of growth factors such as insulin-like growth factor (IGF) (242). After delivery, there is a rapid decline in the levels of pregnancy hormones and maternal physiology rapidly returns to normal, although during lactation the levels of prolactin and oxytocin are elevated (243).

Immune changes in pregnancy tend to suppress the maternal immune system; this is thought to be important in preventing rejection of the fetus, which contains foreign (paternal) antigens (244–246). During pregnancy, there are increased Th2 responses and reduced Th1- and Th17- responses (237, 247) and increased levels of circulating Treg cells (186, 248, 249). There are also increased Treg cell numbers in the placenta and draining lymph nodes (250–252). Other evidence of increased Treg activity in pregnancy comes from findings of increased foxp3 expression and increased functional suppression in pregnant and estrogen treated rats (253). The increased level of Treg cells in pregnancy is thought to be due to the effects on the immune system of E2 (185). The changes in pregnancy also include a shift to Th2 immune responses and a shift to greater humoral than cell mediated immunity (254). B cells also show changes in pregnancy, with increases in numbers of regulatory B cells (255).

### Effects of pregnancy on incidence and severity of EAE

The effects of pregnancy on EAE are summarized in **Table 8**. In general, pregnancy reduces the incidence and severity of disease in all models at some time point; however, the specific details of when the effects were most notable differ, depending on the timing of EAE induction vs the beginning of pregnancy.

**Table 8:** Effects of pregnancy in EAE.

Antigen	Animal strain	Time of induction of EAE	Effect	References
Guinea pig spinal cord	Guinea pigs (gestation time 63 days)	During pregnancy (from day 16-57)	Delayed onset and reduced severity at all time points vs non-pregnant. <50% of pregnancies went to full-term. Guinea pigs that underwent spontaneous abortion or resorption developed EAE 2-3 days after foetal rejection.	(256)
Guinea pig spinal cord	Lewis rats (gestation time 21-23 days)	During pregnancy (from day 2 to day 20)	Delayed onset of EAE. Rats with EAE induced during days 17 to 20 were most protected (7/12 did not develop EAE). No pregnant rats aborted or resorbed foetuses.	(256)
Guinea pig spinal cord	Lewis rats	During pregnancy	Rats inoculated in second and third week had delayed EAE onset. Those inoculated during week 3 had decreased severity of disease.	(257)
Bovine brain	DA rats	During pregnancy (first, second or third week)	Reduced severity in group immunized during 2^nd^ week; others had delayed onset, but increased disease severity after delivery.	(258)
Bovine brain	Rabbits (gestation time 31-32 days)	During pregnancy (between days 5-15 or 20-25)	Delayed onset and disease incidence; effect most notable in rabbits immunized during days 20-25	(259)
Guinea pig MBP	Lewis rats	Day 1 after mating	Reduced severity, day of onset of EAE same as non-pregnant controls. More IL4, IL10 and TNF produced by pregnant vs non-pregnant rats	(260)
PLP_139-151_	SJL/J mice (gestation time 21 days)	During pregnancy (between days 2-7, 8-13, 14-16)	Reduced incidence of EAE when immunized at either time point. EAE induced during days 8-13 led to large number of resorptions and aborted pregnancies. No decrease in severity in mice that developed EAE.	(261)
PLP_139-151_	SJL/J mice	During pregnancy (between days 11-13, 15-18)	Reduced incidence of EAE when EAE induced during days 15-18. Increased IL10, and decreased IL17 and TNFα at this timepoint.	(262)
MOG_35-55_	C57BL/6	During late pregnancy (days 16-18)	Reduced severity	(262)
PLP_139-151_	SJL	Pregnancy just after the peak of first attack of EAE	Disease less severe during pregnancy, especially during late pregnancy, but increased to same level as virgin control mice after delivery	(263)
MOG_35-55_	C57BL/6	Pregnancy just after the peak of EAE	Disease less severe during pregnancy, but after pregnancy mice did not increase to the same level of severity as virgin controls	(263)

In guinea pigs, which have a much longer gestation period (63 days) than mice and rats (~21 days), induction of EAE at any timepoint during pregnancy delays the onset of disease and reduces the severity of EAE compared to induction of disease in non-pregnant animals (256). In rabbits (31-32 days gestation), pregnancy suppresses EAE, with the effects most noticeable when EAE is induced during mid to late pregnancy (day 20-25) (259). Similarly, Lewis rats with EAE induced during the third weeks of pregnancy show the greatest level of protection against the development of disease; rats inoculated in the first week or second week of pregnancy are less protected (256, 257). Interestingly, however, when those rats are re-challenged again 3 weeks after weaning of their litters rats that were protected in pregnancy are more susceptible to the second encephalitogenic challenge, suggesting either that the protective effects during pregnancy are not antigen specific, or that they are not very long-lasting (257). In DA rats, EAE is less severe when disease is induced during the second week of pregnancy, although the rats develop more severe relapses post-partum (258).

In various mouse models, active induction of EAE during late pregnancy leads to a reduction in the incidence of EAE and/or a decrease in clinical severity, compared to virgin controls (261, 262). In contrast, adoptive transfer of activated MBP-specific T cells into pregnant mice during mid-pregnancy, but not in late pregnancy, resulted in a delayed onset of EAE and reduced clinical severity in comparison to virgin controls (264).

The effects of becoming pregnant after the onset of EAE have also been investigated. In both SJL mice with PLP-induced EAE and in C57BL/6 mice with MOG-induced EAE, onset of pregnancy leads to clinical improvement of EAE, but the pathological lesions persist (263). In mice followed until after delivery, EAE scores in the SJL mice return to the same level as in virgin controls, whereas C57BL/6 mice continue to have milder disease than the virgin controls (263).

There have been attempts to transfer the protective effects of pregnancy. Offspring from rats that had EAE during pregnancy show transient protection from EAE, and rats suckled by mothers that had EAE in pregnancy also acquire protection (265).

### Effects of pregnancy on EAE pathophysiology

Several potential mechanisms have been put forward to explain the beneficial effects of pregnancy on EAE. There are alterations of cytokine production (increased expression of mRNA for IL-4 and IL-10) in the spinal cords of pregnant compared to non-pregnant Lewis rats with EAE induced one day after mating, and serum from pregnant rats suppresses lymphocyte proliferation (260, 261). A suppressive serum factor found in late pregnancy has also been implicated in the amelioration of EAE in SJL mice when EAE is induced during the second or third week of pregnancy. Mice with EAE induced during the first week of pregnancy are not protected, suggesting that the serum factor acts on the induction and early effector phases of EAE (261).

Exosomes from both pregnant and non-pregnant mice suppress EAE. However, exosomes are more abundant in pregnancy, and it is suggested that they contribute to protection during pregnancy by suppressing T cell activation and promoting maturation of oligodendrocyte precursor cells and their migration into CNS lesions (266).

Other studies have shown that pregnancy does not appear to affect the induction of myelin-specific T cells, and that there is actually an increase in numbers of such cells in the spleen compared to non-pregnant mice; however, these T cells produce less TNF and IL-17 than those from non-pregnant mice, and there is a higher frequency of IL-10-secreting cells within the CD11b^+^, CD11c^+^, CD19^+^, and CD4^+^/CD25^+^ Treg populations (262). Treg cells bearing TCR clonotypes related to the encephalitogenic TCR are increased in pregnant mice in which EAE has been induced on day 7 of pregnancy, whereas such cells are not seen in virgin controls (267). These results suggest that when an antigen is introduced during pregnancy, an immunoregulatory environment towards that antigen predominates.

Although pregnancy is a state when there are circulating growth factors, and although estrogen and progesterone are neuroprotective, there is little information about the effects of pregnancy on neuroprotection and remyelination in EAE.

### Effects of other pregnancy related molecules

Several other factors that could contibute to the suppression of EAE by pregnancy have been identified, but only a limited number of studies has been done on each of them. They include:

#### Early pregnancy factor (EPF)

EPF was first identified as a factor appearing in the serum shortly after conception (268). EPF is identical to chaperonin 10 (269, 270). It is immunomodulatory and has some growth factor-like properties, including enhancing survival of oligodendrocyte precursors in culture (271, 272). EPF suppresses EAE in Lewis rats and in SJL/J mice (273–276).

#### Alpha-fetoprotein

Alpha-fetoprotein, a glycoprotein produced by the fetus during pregnancy, has immunomodulatory properties (277). In guinea pigs, alpha-fetoprotein administered daily from the time of induction of EAE is able to prevent the development of clinical signs of EAE, and treatment with alpha-fetoprotein commencing after onset of disease also results in a decrease in disease severity (278). In rabbits, treatment with alpha-fetoprotein (daily injections after the onset of clinical signs) also suppresses disease (279). In female C57BL/6 mice with MOG-induced EAE and treated with human alpha-fetoprotein there is increased expression of apoptosis genes and reduced severity of disease (280).

#### Interferon-tau (IFN-τ)

IFN-τ is a type I interferon that is produced in ruminants during pregnancy, and binds to the same receptors as other type I interferons (281). Oral administration of IFN-τ suppresses MBP-induced EAE in NZW mice (282) and both oral and intra-peritoneal administration of IFN-τ after the onset of disease suppresses relapses of EAE induced by MBP in SJL/J mice (283).

#### Insulin-like growth factor

Insulin-like growth factor (IGF) has immunomodulatory and immunosuppressive effects (284) and is present in increased levels in serum during pregnancy (242). IGF treatment reduces the severity of EAE in Lewis rats (285).

#### Pre-implantation factor (PIF)

PIF is a peptide found in the maternal circulation (286). It has a role in immune modulation and neuroprotection (287), and a synthetic version of PIF can reduce signs of PLP-induced EAE in SJL mice, *via* induction of changes to the phosphorylation state of many molecules in the brain (288).

### In summary

Overall, pregnancy (particularly the latter part) is a highly immunosuppressive state in almost every EAE model, and studies of pregnancy across different models show more consistent results than do other studies of the effects of sexual dimorphism in EAE. The effects of pregnancy appear to be driven largely, but not solely by estrogen levels, but there are also numerous other pregnancy-related molecules that have been identified that likely contribute to the overall effects of pregnancy. Further study of these molecules is warranted.

## Conclusions

Many studies provide evidence that biological sex and pregnancy influence the clinical course of EAE. EAE is a complex disease that has an induction phase involving immune cells in the periphery, an effector phase when the immune cells move into the CNS and when the ability of the nervous system to both take part in the inflammatory response and also to resist damage plays a role, and a recovery phase, when restorative and neuroprotective processes play a role. As indicated in our review, sex- or pregnancy-determined effects can affect each stage of EAE, and their overall effects reflect the net effect of sex differences in many aspects of the pathophysiology of EAE. Additionally, there are other intrinsic and extrinsic factors that can modify the course of EAE and that can also be affected by biological sex. Importantly, the effects of sex vary from one animal model to another and are complex and cannot be widely extrapolated. In EAE, it is therefore essential that studies looking at the effects of biological sex or pregnancy provide full information about the model that is used (i.e. animal strain, sex, the inducing antigen, timing of EAE induction in relation to pregnancy etc.) and, preferably, that more than one EAE model is used to show if any observed effects are generalizable.

There is some evidence of the underlying mechanisms by which sex and pregnancy alter the pathogenesis of EAE. **Tables 4** and **5** list the many cell types and cellular processes that have been implicated thus far. These studies show that sex differences in EAE can be found at all stages of disease (induction stage, effector stage, recovery stage), in many cell types and in many molecules and pathways. However, due to the aforementioned complexity and variability from one model to another, studies that comprehensively identify the full gamut of mechanisms underlying the effects of sex or pregnancy on EAE are lacking and some of the effects are likely to be specific to different animal strains. However, despite the variability amongst studies, there are clearly effects of sex and pregnancy in EAE, likely due to the effects of sex hormones and sex chromosomes, and these effects could all be relevant to MS. Going forward, we suggest that even though there is variability among animal strains species and, molecules and pathways that show sex differences are likely to be important in disease. To study these more thoroughly will require investigators to be mindful of the possibility of sex differences and to consider adding additional control groups to experiments. Because the effects of sex hormones vary with age, it would also be useful to study young, adult and aged animals. This is clearly a field that requires further work in order to better our understanding of the mechanisms of sex differences, which are fundamental to biology, and to suggest possible targets or therapies for MS.

## Author contributions

PM and JG conceived the idea, conducted literature searches, and drafted the manuscript. Both authors approve the manuscript for publication.

## Funding

Funded by Grant 1164036 from the National Health and Medical Research Council, Australia.

## Conflict of interest

The authors declare that the research was conducted in the absence of any commercial or financial relationships that could be construed as a potential conflict of interest.

## Publisher’s note

All claims expressed in this article are solely those of the authors and do not necessarily represent those of their affiliated organizations, or those of the publisher, the editors and the reviewers. Any product that may be evaluated in this article, or claim that may be made by its manufacturer, is not guaranteed or endorsed by the publisher.

## Abbreviations

BBB, blood–brain barrier; bSCH, bovine spinal cord homogenate; DC, dendritic cells; E2, estradiol; E3, estriol; EAE, experimental autoimmune encephalomyelitis; EPF, early pregnancy factor; ERα, estrogen receptor alpha; FCG, four core genotype; GM-CSF, granulocyte/macrophage colony stimulating factor; gpSCH, guinea pig spinal cord homogenate; IFNγ, interferon gamma; IFNτ, interferon tau; IGF, insulin-like growth factor; IL, interleukin; ILC2, innate lymphoid cell type 2; LPS, lipopolysaccharide; M-CSF, macrophage colony stimulating factor; MBP, myelin basic protein; MOG, myelin/oligodendrocyte glycoprotein; MS, multiple sclerosis; NK, natural killer; PPAR-γ, peroxisome proliferator-activated receptor gamma; PIF, preimplantation factor; PLP, myelin proteolipid protein; S1PR2, sphingosine-1-phosphate receptor 2; TCR, T cell receptor; TGF-β, transforming growth factor beta; TNF, tumor necrosis factor; Treg, regulatory T cell; VLA-4, very late antigen 4 (integrin α4β1).
